# Alternative and Complementary Approaches to Consider for Effective *Babesia* Vaccine Development

**DOI:** 10.3390/pathogens12091166

**Published:** 2023-09-16

**Authors:** Jennifer Maye, Alejandro Cabezas-Cruz

**Affiliations:** 1 SEPPIC Paris La Défense, 92250 La Garenne Colombes, France; 2 ANSES, INRAE, Ecole Nationale Vétérinaire d’Alfort, UMR BIPAR, Laboratoire de Santé Animale, 94700 Maisons-Alfort, France

The *Babesia* genus encompasses several species of apicomplexan hemoprotozoan parasites. These parasites, primarily transmitted by ticks, have gained attention due to their impact on the health of animals and humans. The significance of babesiosis, the disease caused by *Babesia* parasites, has grown substantially in recent years, with its classification as an emerging zoonotic disease, and the prevalence of human cases has been on the rise [1,2].

The *Babesia* life cycle involves asexual stages in vertebrate hosts and sexual stages in ticks. The complex interplay between hosts and vectors complicates the development of effective control strategies, especially considering the lack of vaccines and the challenge of drug resistance [3,4,5]. Babesiosis presents with a range of symptoms, from mild to severe, and can be especially dangerous for immunocompromised individuals, making the development of preventive measures a priority [6,7,8,9].

Developing anti-Babesia vaccines is imperative. While vaccines against human [10] and bovine [11] babesiosis have been advanced, current research and development efforts face several hurdles. A recent review by Jerzak et al. [12] sheds light on the complexities of developing an effective *Babesia* vaccine. It emphasizes the strengths and limitations of current vaccine candidates, including whole-pathogen and subunit vaccines. The review also highlights the importance of adjuvant selection for effective vaccine development.

There are many challenges associated with whole-parasite vaccines, including the risk of contamination during manufacturing, the difficulty in maintaining *Babesia* parasites in continuous culture, the potential reversion to virulence in attenuated vaccines, and the storage and deployment issues that result due to the presence of intact RBC membranes [12]. In contrast, protein-based subunit vaccines face challenges related to the genetic diversity of *Babesia* species [10,13,14], uncertainty about the role in cytoadherence [12], and specificity of epitopes required for effective protection [12]. Notably, a common challenge faced by both types of vaccines revolves around the choice and development of suitable adjuvants.

Adjuvants are substances that enhance the immune response to antigens, and their importance in vaccine development cannot be overstated [15,16,17,18]. Adjuvants play a pivotal role in guiding the immune system's reaction to vaccine antigens, which influences the type and strength of immune responses generated [15,16,17,18]. Critical areas for improvement in *Babesia* vaccines encompass the development of adjuvants tailored to specific species, the optimization of adjuvant formulations to boost immune response and enhance vaccine effectiveness, as well as the selection of adjuvants suitable for clinical applications [16].

Whole-parasite vaccines have shown promise, as they present all antigens of the parasite and reduce the impact of antigenic diversity. Indeed, a whole-parasite vaccine using red blood cells parasitized with the human parasite *Babesia microti* provided heterologous protection, was effective in splenectomized animals, and could be lyophilized for extended shelf life [10]. This marks a significant advancement towards a potential human vaccine trial. Moreover, it is worth noting that whole-parasite vaccines for bovine babesiosis already exist [19]. In contrast to subunit vaccines that rely on antibodies to block infection, whole-parasite vaccines activate CD4+ T cells and macrophages, providing a different mode of protective immunity. Activation of CD4+ T cells in the *B. microti* vaccine occurred in the absence of adjuvants [10]. The authors of the study proposed that this could be due to the presence of parasite antigens encased within a membrane, which would closely mimic natural infection [10]. However, a high dose of parasite per vaccine, typically equalling or exceeding the million parasite or parasite equivalents per vaccine dose [10,20], is required in whole-parasite inactivate vaccines. This low immunogenicity could be overcome using appropriate human-compatible strong adjuvants [10,20].

Efforts to develop subunit vaccines for combating *Babesia* infections have involved the identification and characterization of specific protein-based antigens. Babesia merozoite has emerged as a primary candidate for subunit vaccine development due to the challenges associated with sporozoite-based vaccines and the potential for different efficacy levels against various *Babesia* species [12]. Several *Babesia* merozoite surface antigens have been explored as potential subunit vaccine candidates. These include BMSA, BmSA1, BmSP44, methionine aminopeptidases, heat shock protein 70, and PROF [12]. These antigens have been shown to elicit antibody responses and reduce parasitemia in animal models, making them promising candidates for future vaccine development. However, the selection of appropriate adjuvants in vaccine development plays a pivotal role in overcoming several challenges associated with vaccine efficacy and safety [16]. Attenuated and inactivated vaccines, while effective, often have limitations such as low yield, long culture cycles, and safety concerns. Subunit vaccines with improved safety profiles often lack strong immunogenicity when used alone. Adjuvants are essential in enhancing immune responses and increasing vaccine efficacy. For instance, the recombinant subunit vaccine Shingrix®, formulated with the Adjuvant Systems 01 (AS01), has demonstrated superior effectiveness compared to the live attenuated vaccine Zostavax® for preventing shingles [21,22,23]. Adjuvants facilitate improved antigen presentation, leading to long-lasting protection against pathogens.

In general, adjuvant selection in *Babesia* vaccine research deserves careful consideration. For instance, when a recombinant form of the *B. microti* protein methionine aminopeptidase 1 (rMetAP1) was administered to mice along with complete Freund's adjuvant, it resulted in a reduced parasitemia [24], yet this adjuvant is incompatible for human use. Similarly, another subunit vaccine based on the major surface antigen of *B. microti* merozoites (BMSA) elicited a significant antibody-mediated inhibition of parasite invasion but relied on the same unsuitable Freund's adjuvant [25]. This highlights the challenge of finding adjuvants that are not only effective but also safe for human use in *Babesia* vaccine development.

Recent research highlights a complementary approach to classical *Babesia* vaccines. Ticks, the vectors responsible for *Babesia* transmission, harbor a complex microbiota that can influence both vector physiology and vector competence [26]. Targeting keystone taxa of tick microbiota [27] through anti-microbiota vaccines has been shown to alter tick feeding behavior [28] and modulate the composition of bacterial communities in the vectors [29]. This approach affected the development of the tick-borne bacterial pathogen *Borrelia afzelii* [30] and the mosquito-borne apicomplexan parasite *Plasmodium relictum* [31] in the vector, and thus it holds promise for *Babesia* and other vector-borne pathogens [32]. By perturbing the microbiota through anti-microbiota antibodies [32], *Babesia* stages different from merozoite and including zygote, ookinetes, or sporozoites can be indirectly affected in the vector, potentially reducing transmission rates. This innovative strategy underscores the link between vector biology and pathogen transmission, complementing vaccine efforts.

In order to maximize the impact of these strategies, it is imperative to bridge the gap between immunology and microbiome research. Collaborative efforts between researchers, immunologists, parasitologists, and microbiome experts are essential to harness the potential of anti-microbiota vaccines in tandem with conventional vaccine development for *Babesia* and other vector-borne diseases. Moreover, the ongoing exploration of novel adjuvants can further enhance the efficacy of both traditional and innovative vaccine candidates.

In conclusion, advancing adjuvant research is essential in the quest for an effective *Babesia* vaccine. The review article by Jerzak et al. [12] emphasizes the intricate challenges of preventing *Babesia* infections and highlights the potential of various vaccine candidates along with the need to innovate on adjuvants development. Moreover, recent findings in microbiome research suggest that anti-microbiota vaccines could complement traditional vaccine efforts by targeting tick vectors and their microbiota. Through collaboration and innovation, researchers can create a safer future for animals and vulnerable humans by preventing *Babesia* infections.
